# Hydrogen embrittlement through the formation of low-energy dislocation nanostructures in nanoprecipitation-strengthened steels

**DOI:** 10.1126/sciadv.abb6152

**Published:** 2020-11-11

**Authors:** P. Gong, J. Nutter, P. E. J. Rivera-Diaz-Del-Castillo, W. M. Rainforth

**Affiliations:** 1 Department of Materials Science and Engineering, Sir Robert Hadfield Building, Mappin Street, Sheffield S1 3JD, UK.; 2 Department of Engineering, Engineering Building, Lancaster LA1 4YW, UK.

## Abstract

Hydrogen embrittlement is shown to proceed through a previously unidentified mechanism. Upon ingress to the microstructure, hydrogen promotes the formation of low-energy dislocation nanostructures. These are characterized by cell patterns whose misorientation increases with strain, which concomitantly attracts further hydrogen up to a critical amount inducing failure. The appearance of the failure zone resembles the “fish eye” associated to inclusions as stress concentrators, a commonly accepted cause for failure. It is shown that the actual crack initiation is the dislocation nanostructure and its associated strain partitioning.

## Teaser

Embrittlement originates from dislocation nanocellular structures stabilized by the presence of hydrogen.

## INTRODUCTION

Embrittlement due to the presence of hydrogen has been reported for over a century. From the seminal work of Johnson (*1*), it has been recognized that ductility can be markedly decreased upon the formation of interfacial chemical compounds of which hydrogen can be a by-product (*2*). Hydrogen can then diffuse to the component bulk, producing a wealth of interactions at various scales with the microstructure fostering the formation and propagation of cracks (*3*, *4*). Hydrogen embrittlement (HE) is recognized to have a marked effect in emerging technologies; these include wind turbines for electricity generation, hydrogen storage, and ultralight car bodies (*5*, *6*). This makes HE a prominent issue of great importance in modern societal and technological needs.

At the center of HE scientific discussions is the hydrogen-microstructure interaction in advanced alloys such as steels. Rotating heavily loaded components such as gears and bearings in wind turbines undergo accelerated damage due to hydrogen, which, upon interaction with strengthening precipitates such as cementite, promote their dissolution, accelerating crack formation (*7*). Twinning-induced plasticity (TWIP) steels display strong hydrogen-twin interactions, where the twins become the medium for rapid hydrogen diffusion, which, upon accumulation at certain regions, promote void formation and cleavage (*8*, *9*). Embrittlement in landing gear for aircraft is thought to be induced when hydrogen gathers around inclusions, promoting further accelerated damage of the ultrahard martensitic structure (*8*). Super duplex stainless steels for hydrogen storage display preferential crack formation in ferrite, from where brittle fracture starts once a critical hydrogen concentration is reached (*10*, *11*). The wide range of microstructures in which hydrogen promotes failure have one thing in common: a complex microstructure with well-engineered structures controlling strength, ductility, and toughness. Such features spatially array lattice strains that direct dislocation motion on deformation; as illustrated by the aforementioned examples, such arrays as well as the dislocation movement are altered by the presence of hydrogen (*12*, *13*). Thus, the excellent properties of sophisticated microstructures become ameliorated by hydrogen, diminishing component performance. The problem is of great importance as HE failures have substantial financial impact.

Several solutions to HE have been proposed. Controlling the component environment or inhibiting hydrogen ingress through coatings are usual solutions. These are, however, difficult to realize; for instance, the offshore environment of wind turbines remains fixed and lubricant decomposition would require substantial alteration of its chemistry (*14*, *15*). Coatings applied to rolling elements in wind turbines may alter their rolling contact performance, and their wear has to be controlled (*16*, *17*). It follows that a common solution is altering the component bulk microstructure, which can be achieved through the addition of certain elements, which, in solid solution in the matrix, may alter HE susceptibility, e.g., Al in TWIP steels (*18*) or Cr in precipitation hardening steels (*19*). However, such additions do not fully preclude HE, so hydrogen trapping has been a common alternative solution. Hydrogen is attracted to microstructural features such as nanoprecipitates, bulk phases, or interfaces. Such attraction gathers hydrogen in prescribed positions where its ability to gather and produce damage is substantially reduced. Nanoprecipitates, such as (Nb,V,Ti)C, can attract hydrogen either to their strain fields or the bulk (*20*, *21*); in either case, the energy required for it to go back into solid solution in the matrix and recombine inducing damage is higher than that to stay trapped at room temperature. As such, the nanoprecipitates are spread across the microstructure and, upon hydrogen ingress, atomic hydrogen becomes immobilized throughout. Alternatively, phases such as austenite display a substantially lower diffusivity yet higher solubility to hydrogen, potentially acting as barriers to hydrogen diffusion when evenly spread across the microstructure (*22*, *23*). Carefully engineered interfaces of prescribed orientation relationships and controlled strain fields, such as the interface between film retained austenite and bainite in nanostructured bainite, may also be a potent hydrogen trap, especially when evenly spread across the microstructure in slowly transformed material (*24*). Those solutions to HE are just palliatives that only delay failure, not fully eliminating the problem but often delaying it beyond the expected life span of the component.

Failure due to hydrogen has usually been related to three distinct types of mechanisms: hydrogen-enhanced decohesion, wherein the binding force is decreased promoting cleavage across crystallographic planes (*25*, *26*); hydrogen-enhanced strain-induced vacancy formation, appearing when clusters of vacancies are stabilized in the presence of hydrogen, growing to a critical size consistent with crack growth, and leading to failure (*27*, *28*); and hydrogen-enhanced localized plasticity, which has received much attention in the literature. It assumes that hydrogen increases the mobility of dislocations emitted from the tip of a growing crack, increasing its growth rate (*29*, *30*). Multiple researchers have suggested mixed mechanisms, where a crack forms under a single mechanism followed by propagation under an alternative one.

**Fig. 1:**
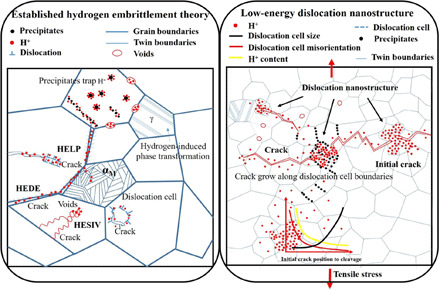
Multiscale depiction of HE. (**Left**) Accepted theories of HELP (hydrogen-enhanced localized plasticity), with strong interaction with dislocations at crack tips; HESIV (hydrogen-enhanced strain-induced vacancy formation), forming clusters of vacancies (voids) at the tips; and HEDE (hydrogen-enhanced decohesion), promoting decohesion. (**Right**) New mechanism proposed in this work. Hydrogen diffuses to crack tips, where its concentration increases, promoting dislocation cell formation, which, upon reaching a critical level, causes failure.

The myriad of possible hydrogen-microstructure interactions is illustrated in Fig. 1, where atomic hydrogen (H^+^) ingress is followed by interactions with dislocations (⊥), promoting transgranular and intergranular cracks, leading to the formation of voids or twins, while interacting with phases such as austenite and precipitates. The main purpose of this work is to disentangle the fundamentals of HE by examining one of the simplest possible microstructures, a low-alloy ferritic grade with a fine structure of nanoprecipitates. The examination is carried out by comparing deformation mechanisms in the presence and absence of hydrogen, at scales ranging from nanometers to millimeters. We find that low-energy dislocation cell structures are responsible for crack initiation, and growth is controlled by brittle fracture.

## MATERIALS AND METHODS

Two steel grades of composition shown in Table 1 are considered, hereafter termed Ti-Mo and V-Mo, respectively, aimed at the interphase precipitation of titanium carbide (TiC) and vanadium carbide (VC). Interphase precipitation occurs as ferrite forms from austenite during an isothermal hold. In this case, a two-step heat treatment was used, consisting of preheating to 1250°C for 30 min and water quenching, followed by firstly heating at a rate of 10°C/s to 1200°C, where the alloys were held 3 min and further cooled to 630°C at a rate of 10°C/s, where they were isothermally transformed for a period of 90 min (when interphase precipitation takes place) before water quenching to room temperature.

**Table 1:** Chemical composition of the experimental steels (wt %).

**Material**	**C**	**Si**	**Mn**	**Al**	**V**	**Ti**	**N**	**Mo**
Ti-Mo	0.1	0.2	1.6	0.045	—	0.2	≤10 ppm	0.5
V-Mo	0.1	0.2	1.6	0.045	0.2	—	≤10 ppm	0.5

To replicate hydrogen ingress, specimens for tensile testing were electrochemically charged into using 1 g/liter in an aqueous solution of 3 weight % (wt %) NaCl and 0.3 wt % NH_4_SCN with a current of 10 mA cm^−2^ for 48 hours at room temperature. Slow strain rate tensile tests were then conducted at a constant strain rate of 10^−5^ s^−1^ at room temperature. Microstructures of tensile fractures from two steels were examined using a field-emission scanning electron microscope (FESEM). Transmission electron microscope (TEM) thin foils were extracted from site-specific locations of the fracture surface using the focused ion beam (FIB) lift-out technique with the FEI Helios Nanolab 650 SEM/FIB instrument, enabling the examination of deformation microstructures immediately beneath the fracture surface. During the FIB lift-out from the fracture surface, platinum (Pt) was slowly deposited on the location of interest to preserve the corresponding fracture surface at the location and the microstructure below it. TEM observation of the thin foils was then conducted in the JEOL F200 TEM operated at an accelerating voltage of 200 kV. In all cases, imaging was carried out in scanning TEM mode so as to show the highest dislocation density.

## RESULTS

Thermal desorption analysis (TDA) (*31*) showed the V-Mo to trap a higher amount of hydrogen of 0.144 compared to 0.101 weight parts per million (wppm) for Ti-Mo, a behavior consistent with lattice parameters and mechanical behavior to be presented next. Conversely, dislocations in V-Mo trap 0.286 compared to 0.436 wppm in Ti-Mo, which is consistent with the enhanced plasticity shown in V-Mo.

**Fig. 2:**
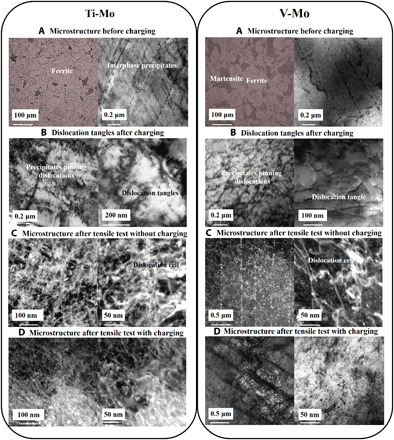
SEM and TEM micrographs showing the microstructure before charging and dislocation and precipitate structures after charging. Ti-Mo and V-Mo (**A**) before and (**B**) after charging, and (**C**) after tensile testing with no and (**D**) with hydrogen charging.

High-resolution TEM was performed on both hydrogen-free and -charged specimens. Figure 2 shows representative images of Ti-Mo and V-Mo microstructures before charging (Fig. 2A), after charging (Fig. 2B), after tensile test in hydrogen-free specimens (Fig. 2C), and after tensile test with charging (Fig. 2D). Figure 2A shows for both grades a ferritic microstructure with patches of martensite, but fine parallel lines of TiC and VC interfacial precipitates, respectively, for the Ti-Mo and the V-Mo alloy. The average precipitate radius and number density were quantified using the replica method and are listed in Table 2. Immediately after charging, and in the absence of an external stress, spontaneous dislocation tangles were formed as shown in Fig. 2B. The cell sizes are coarser in V-Mo, and the corresponding values are shown in Table 2. Upon deformation, fine dislocation structures are formed as depicted in Fig. 2C for hydrogen-free specimens, but even finer once deformation has taken place in hydrogen-charged specimens (Fig. 2D). Owing to the increased number of dislocation interactions with strain, especially around precipitates, dislocation density increases with strain. However, upon detailed microstructural inspection, a unique effect is observed; the formation of low-energy dislocation nanostructures, which were observed to be at the origin of cracks, are hereon termed dislocation nanostructures.

**Table 2:** Quantitative metallography.

**Material**	**Dislocation** **density (10^13^ m^−2^)**	**Precipitate density** **(μm^−2^)**	**Precipitate radius** **(nm)**	**Dislocation cell** **size (nm)**	**Dislocation** **misorientation (°)**	**Residual stress** **(MPa)**
Ti-Mo uncharged	5.02	550	5.8	120	1–5	−343
Ti-Mo charged	23.9	—	—	177	15–35	−602
V-Mo uncharged	3.96	420	7.9	144	1–7	−347
V-Mo charged	11.6	—	—	193	40–60	−470

**Fig. 3:**
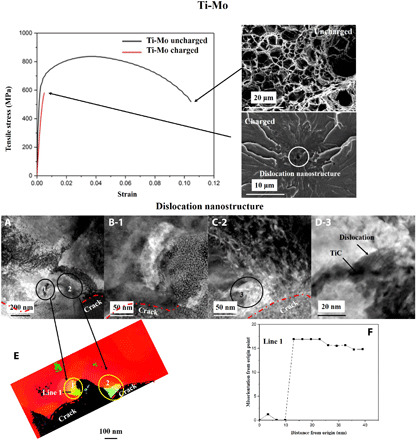
Ti-Mo mechanical response in the fractured region in hydrogen-free and -charged specimens. (**A**) FIB lamella taken from dislocation nanostructure, highlighting regions 1 and 2, respectively shown in (**B**) and (**C**). (**D**) Region 3 showing TiC/dislocation interaction. (**E**) Misorientation map around regions 1 and 2, with the corresponding misorientaiton values of line 1 in (**F**).

**Fig. 4:**
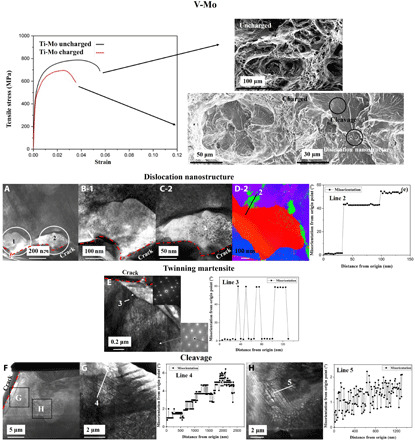
V-Mo mechanical response in the fractured region in hydrogen-free and -charged specimens. (**A**) FIB lamella taken from the dislocation nanostructure showing heavily deformed regions 1 and 2 around the crack source, respectively, shown in (**B-1**) and (**C-2**). (**D-2**) shows the heavy misorientation mapped in (C-2). The twin martensite induced by hydrogen is shown in (**E**). Regions away from the crack source are shown in (**F**), in (**G**) and (**H**) where modest misorientation serrations are shown in the cleavage region.

The crack dislocation nanostructures display unique features revealed in Fig. 3 (Ti-Mo) and Fig. 4 (V-Mo). Referring to Fig. 3, a very pronounced decrease in ductility for the charged specimen is shown, with failure occurring at the onset of plastic deformation. Fracture originates from the highlighted dislocation nanostructure present in the charged specimen, whereas a dimple ductile structure is appreciated in the uncharged counterpart, consistent with the uncharged stress–strain curve. FIB lamellae of the crack origin in the charged specimen reveal dislocation nanostructures shown in Fig. 3A, with regions 1 and 2, respectively, magnified in Fig. 3 (B and C). Misorientation maps of these areas are shown, indicating typical misorientations ranging from 15° to 17°, as quantified in line 1. The co-operative dislocation/precipitate interactions leading to the increased misorientations is highlighted in region 3 and presented in Fig. 3D.

As for V-Mo shown in Fig. 4, the clear dimple structure of the hydrogen-free specimen is in contrast with the dislocation nanostructure at the center of its hydrogen-charged counterpart. The dislocation nanostructure from which the crack originated shows a cleavage area around it, indicating the mixed character of the fracture zone. First, the very pronounced misorientation around the dislocation nanostructures shown in Fig. 4A, regions 1 and 2, developing around the crack origin are respectively magnified in Fig. 4 (B and C), with the region 2 misorientation map shown in Fig. 4D and the corresponding “line 2” misorientation values in Fig. 4E. Figure 4E shows a remarkably high misorientation of up to 57°. In addition to this, in the regions away from the crack, twin martensite is observed, indicating its corresponding misorientation (line 3). This demonstrates the presence of microstructural changes induced by hydrogen even away from the crack. In contrast to this, the cleaved region shows a stepped misorientation due to the interfacial precipitate fronts, as highlighted by “line 3,” “line 4,” and “line 5,” consistent with the precipitate strain misorientations. The cleaved region therefore is not altered by the hydrogen misorientation as the crack dislocation nanostructure is.

## DISCUSSION

The experimental results demonstrate that Ti-Mo displays less potent traps than V-Mo. Quantitative metallography (Table 2) can give a clear indication of the trapping tendency in terms of the associated lattice strains. Considering a Nishiyama-Wasserman orientation relationship between TiC and VC and the ferrite matrix, the associated lattice strains of each of those carbides is respectively ε_TiC_ = 0.066 compared to ε_VC_ = 0.028. Considering the volume fraction per precipitate of *V*_TiC_ = 0.015 and *V*_VC_ = 0.018 for Ti-Mo and V-Mo, respectively, the nearly equal volume fractions but much lower lattice strains for VC would imply a lower tendency to trapping; however, the TDA results clearly indicate the contrary. This confirms recent atom probe tomography observations that hydrogen is trapped within the precipitate rather at its interface (due to lattice strains) (*20*). Moreover, the early failure in Ti-Mo compared to V-Mo is consistent with dislocations trapping 50% hydrogen in the former than in the latter. Once dislocations are pinned by precipitates, their hydrogen starts to accumulate, producing failure. This is observed in Fig. 3, where strong carbide/dislocation interactions are evidenced.

The most unique aspect of this work is the formation of dislocation nanostructures. Figure 2 demonstrates that, just by charging, dense dislocation tangles are formed. The deformation hydrogen-free specimens promote dislocation cells; these become enlarged in the presence of hydrogen (Table 2). The situation can be modeled using statistical mechanics by computing the energy necessary to form such low-energy dislocation nanostructure (*31*–*33*). The dislocation cell size can be expressed as

$$d_{c}=\frac{6\pi (1-\nu )}{\left(2+\nu \right)}(\frac{1}{2}+\frac{T\;\Delta S_{\text{BCC}}}{2N\mu b^{3}})\frac{1}{\sqrt{\rho }}=\frac{\kappa_{c}^{\text{BCC}}}{\sqrt{\rho }}$$ (1)

where ν is Poisson’s ratio, *T* is the deformation temperature, *N* is the dislocation impingement factor taken as 1 for this calculation, μ is the shear modulus, *b* is the magnitude of the Burgers vector, and ρ is the dislocation density. The dislocation statistical entropy is expressed as

$$\Delta S_{\text{BCC}}=k_{B}\;\text{ln}{\left(\frac{{\overset{\cdot }{\varepsilon }}_{0}+\upsilon }{\overset{\cdot }{\varepsilon }}\right)}^{2}$$ (2)

where *k*_B_ is the Boltzmann constant, ${\overset{\cdot }{\varepsilon }}_{0}$ is the limiting value for the strain rate if the material were deformed at the speed of sound, $\overset{\cdot }{\varepsilon }$ is the deformation strain rate, and υ is the vacancy migration frequency (*34*–*36*). υ showed no substantial influence on the cell size, indicating that the vacancy stabilization effect due to hydrogen bears little influence on the cell structure. Equation 1 stems from energy minimization due to the formation of low-energy dislocation nanostructures of diameter *d*_c_. Another unique feature of hydrogen-assisted failure is the increased misorientation as the crack initiation point is reached, as depicted in Table 2, as well as Figs. 3 and 4. The cell misorientation $\bar{\theta }$ is due to strain energy minimization upon high strain deformation and can be described as

$${\bar{\theta }}^{3}=\frac{3}{2}{\left(\alpha_{\theta }^{\text{BCC}}\right)}^{2}\varepsilon^{2}$$ (3)

where

$$\alpha_{\theta }^{\text{BCC}}=\frac{T\;\Delta S_{\text{BCC}}}{2N\kappa_{c}^{\text{BCC}}\mu b^{3}}$$ (4)

which accounts for the pressure buildup and the cell dislocation nanostructure formation, with a corresponding misorientation and size increase to minimize energy. The application of Eqs. 1 and 3 requires the initial dislocation density and the strain. For the hydrogen-free specimens, the dense precipitate structure constitutes the relevant dislocation nucleation density, as precipitate spacing dictates the formation of dislocation structures; therefore, the precipitate density was input to Eq. 1 for the hydrogen-free conditions, and the measured dislocation density in Table 2 was input. As for the misorientation, the strain values to failure shown in Figs. 3 and 4 were input to Ti-Mo and V-Mo calculations. Consistent with previous research, a dislocation mobility increase of 14 to 17 times produces the accelerated dislocation cell formation. Low-energy dislocation nanostructures characterized by increased misorientation can be well described by gathering 2.5× the amount of hydrogen. The calculation results are shown in Table 3.

**Table 3:** Computation results.

**Material**	**Dislocation cell size** **(nm)**	**Dislocation** **misorientation (°)**
Ti-Mo uncharged	133	1.2
Ti-Mo charged	300	15.2
V-Mo uncharged	153	4.5
V-Mo charged	291	54.5

Upon deformation in a hydrogen-rich environment, complex dislocation-precipitate interactions take place while, simultaneously, cell dislocation nanostructures form. As a result of this, hydrogen at the precipitate/matrix interface may increase dislocation mobility as dislocations approach precipitates; alternatively, diffusion from the precipitate interior to dislocations may take place. Such enhanced dislocation mobility expedites cell formation, although the kinetics of the process cannot be traced. The gliding dislocations synergistically interact with precipitate interfaces and precipitate interiors. Kinetic calculations are required to determine the strain rate dependency of dislocation mobility increase due to hydrogen.

### Summary

A new mechanism for HE is proposed. This is based on ordered strain raisers (e.g., precipitates) controlling dislocation nucleation. Hydrogen ingress immediately produces microstructural changes. It is shown how twinning is induced, as well as the formation of dense dislocation tangles. Hydrogen-rich specimens display enhanced dislocation mobility, fostering the formation of low-energy dislocation nanostructures. The nanostructures can act as sinks for hydrogen, and by gathering an increased amount of hydrogen, their dislocation nanostructures become heavily misoriented originating cracks. Ahead of the crack origin, cleaved areas are visible.

It can be conjectured that when hydrogen is concentrated by crack initiation sites, it is then released. This is consistent with recently observed mechanisms of crack initiation, where carbon nanostructures act as stress risers in 52100 steel subjected to rolling contact fatigue (*37*). Low-energy dislocation nanostructures also form, and their formation is accelerated by the presence of hydrogen (*38*, *39*). Carbon is seen to be rejected from the nanostructured areas, as can be measured in postmortem experiments, although this cannot be established for hydrogen because of its inconspicuous nature.

As a general conclusion, it can be established that microstructures with well-engineered stress raisers will be prone to dislocation nucleation and the formation of low-energy nanostructures, which locally lead to strain partitioning and failure. Whenever a fish eye may have been formed, or in its proximity, the signature of hydrogen can be present through the formation of a low-energy dislocation nanostructure, rather than debonding, void formation, or crack dislocation emission. This demands refocusing the materials scientist’s attention to delaying or inhibiting the formation of hydrogen-assisted dislocation nanostructures.

## Supplementary Material

http://advances.sciencemag.org/cgi/content/full/6/46/eabb6152/DC1

Adobe PDF - abb6152_SM.pdf

Hydrogen embrittlement through the formation of low-energy dislocation nanostructures in nanoprecipitation-strengthened steels

## SUPPLEMENTARY MATERIALS

Supplementary material for this article is available at http://advances.sciencemag.org/cgi/content/full/6/46/eabb6152/DC1
