# Supplementary material for: Hydrogen embrittlement through the formation of low-energy dislocation nanostructures in nanoprecipitation-strengthened steels
Source: Sci Adv. 2020 Nov 11;6(46):eabb6152. doi: 10.1126/sciadv.abb6152 (PMC7673732; doi:10.1126/sciadv.abb6152)
Supplement: http://advances.sciencemag.org/cgi/content/full/6/46/eabb6152/DC1 [file supp_6_46_eabb6152__index.html]

Science Advances | Science AdvancesAAASSearchScience AdvancesMenu

## Supplementary Materials

# Hydrogen embrittlement through the formation of low-energy dislocation nanostructures in nanoprecipitation-strengthened steels

P. Gong, J. Nutter, P. E. J. Rivera-Diaz-Del-Castillo, W. M. Rainforth

Download Supplement

**This PDF file includes:**

- Supplementary Materials and Methods
- Figs. S1 to S6
- References

**Files in this Data Supplement:**

- Adobe PDF - abb6152\_SM.pdf
