# Supplementary material for: Hydrogen embrittlement through the formation of low-energy dislocation nanostructures in nanoprecipitation-strengthened steels
Source: Sci Adv. 2020 Nov 11;6(46):eabb6152. doi: 10.1126/sciadv.abb6152 (PMC7673732; doi:10.1126/sciadv.abb6152)
Supplement: http://advances.sciencemag.org/cgi/content/full/6/46/eabb6152/DC1 [file supp_6_46_eabb6152__1.pdf]

Science Advances
AAAS

advances.sciencemag.org/cgi/content/full/6/46/eabb6152/DC1

Supplementary Materials for

Hydrogen embrittlement through the formation of low-energy dislocation nanostructures in nanoprecipitation-strengthened steels

P. Gong, J. Nutter, P. E. J. Rivera-Diaz-Del-Castillo*, W. M. Rainforth

*Corresponding author. Email: p.rivera1@lancaster.ac.uk

Published 11 November 2020, *Sci. Adv.* **6**, eabb6152 (2020)
DOI: 10.1126/sciadv.abb6152

This PDF file includes:

Supplementary Materials and Methods
Figs. S1 to S6
References

Material production and heat treatment

The chemical composition of two laboratory cast microalloyed steels used in the research containing Ti+Mo (Ti-Mo) and V+Mo (V-Mo) are listed in Table 1. The alloys were made by vacuum induction melting and cast into ingots with dimensions 620 mm × 105 mm × 35 mm at Tata Steel, IJmuiden. The ingots were homogenised at 1250°C for 2 h and hot worked in several passes to 8 mm thick plates. The dilatometer specimens, 120 mm long × 12 mm wide × 6 mm thick, were machined from the plate along the rolling direction. The heat treatment temperature was determined based by the industry coiling temperature for the interphase precipitates, which is between 600-675°C (*40*). Therefore, in this project, the isothermal temperature was determined to 630°C. The aging time was determined based on the dilatometer phase transformation curves shown in Fig. S1. The figure below shows that with aging to 90 min, the austenite phase nearly fully transformed to ferrite phase for both alloys. Therefore, the isothermal aging profile for both Ti-Mo and V-Mo steels is aging at 630°C for 90 min.

Table 1 Chemical composition of the experimental steels (wt%)

**Material**	**C**	**Si**	**Mn**	**Al**	**V**	**Ti**	**N**	**Mo**
Ti-Mo	0.1	0.2	1.6	0.045	-	0.2	≤10 ppm	0.5
V-Mo	0.1	0.2	1.6	0.045	0.2	-	≤10 ppm	0.5

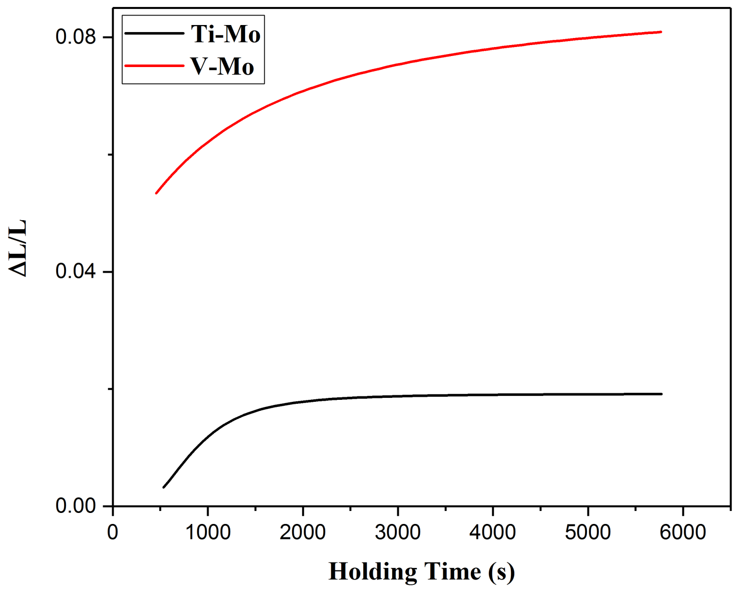

Fig. S1 Dilatation curves of the specimens isothermally treatment at 630°C

The heat treatment for producing interphase precipitated carbides was performed using the Dilatronic dilatometer at The University of Sheffield. Samples were first reheated to 1250°C again, held for 30 min in a tube furnace, and then water cooled to room temperature. The specimens were then austenitized at 1200°C for 3min and cooled to 630°C at a rate of 10°C s^{-1}, held isothermally for 90 min, and finally water quenched to room temperature. The rolling and heat treatment schedule is illustrated in Fig. S2.

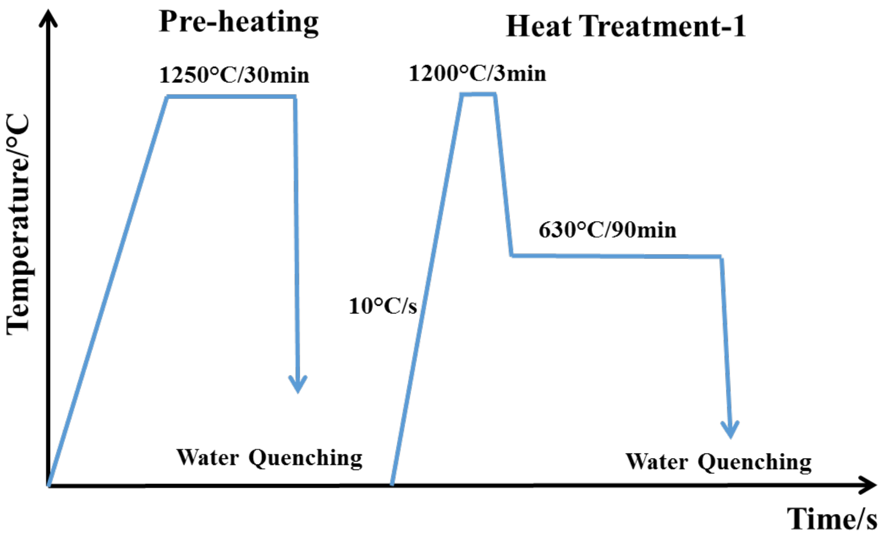

Fig. S2. Schematic of the thermomechanical cycles used and nomenclature used for each condition.

Tensile experiments

Tensile test specimens were prepared according to ASTM standard (E 8M-04) with the long axes parallel to the rolling direction (shown in Fig. S3). Smooth, tensile specimens were 2.66 mm in thickness with a gauge length of 12.5 mm. Slow strain-rate tensile (SSRT) tests were conducted at a constant strain rate of 10^{-5} s^{-1} at room temperature using a Zwick (BTC T1-FR020 TN A50) universal testing machine immediately after charging conditions (*41*, *42*).

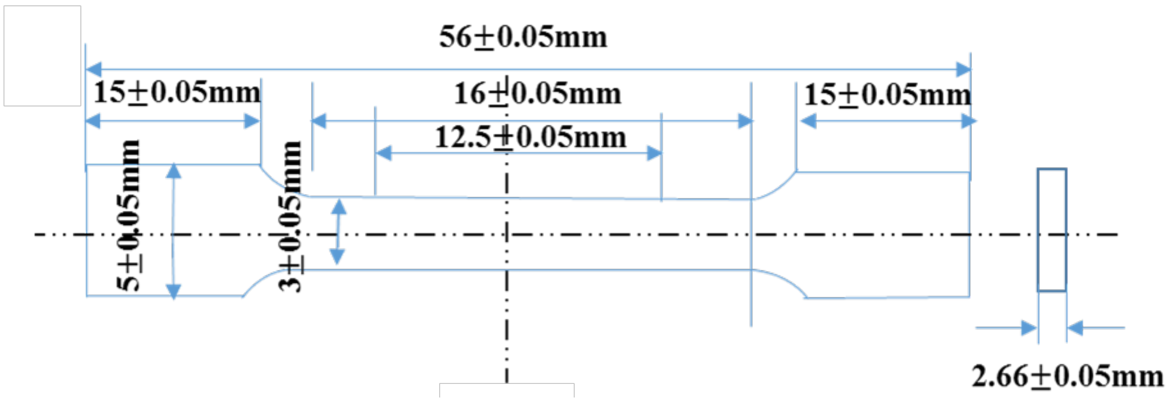

Fig. S3 Tensile sample geometry (lengths in mm).

FIB sample preparation experiments

Microstructures of tensile fractures from two steels were examined using a field-emission scanning electron microscope (FE-SEM). TEM thin foils were extracted from site-specific locations of the fracture surface using the FIB lift-out technique with the FEI Helios Nanolab 650 SEM/FIB instrument, enabling the examination of deformation microstructures immediately beneath the fracture surface, shows in Fig. S4. During the FIB lift-out from the fracture surface, platinum (Pt) was slowly deposited on the location of interest to preserve the corresponding fracture surface at the location and the microstructure below it. After FIB lift out the samples, it has to be transferred to a suitable support micromanipulator for TEM examination. The sample still needs to be thinned for electron transparency to around 200 nm. After that, a low-energy final cleaning has been applied using a Ga+ beam accelerated at 5 keV with milling rate to be 10 nm/min with current of 20-40 pA (*43*).

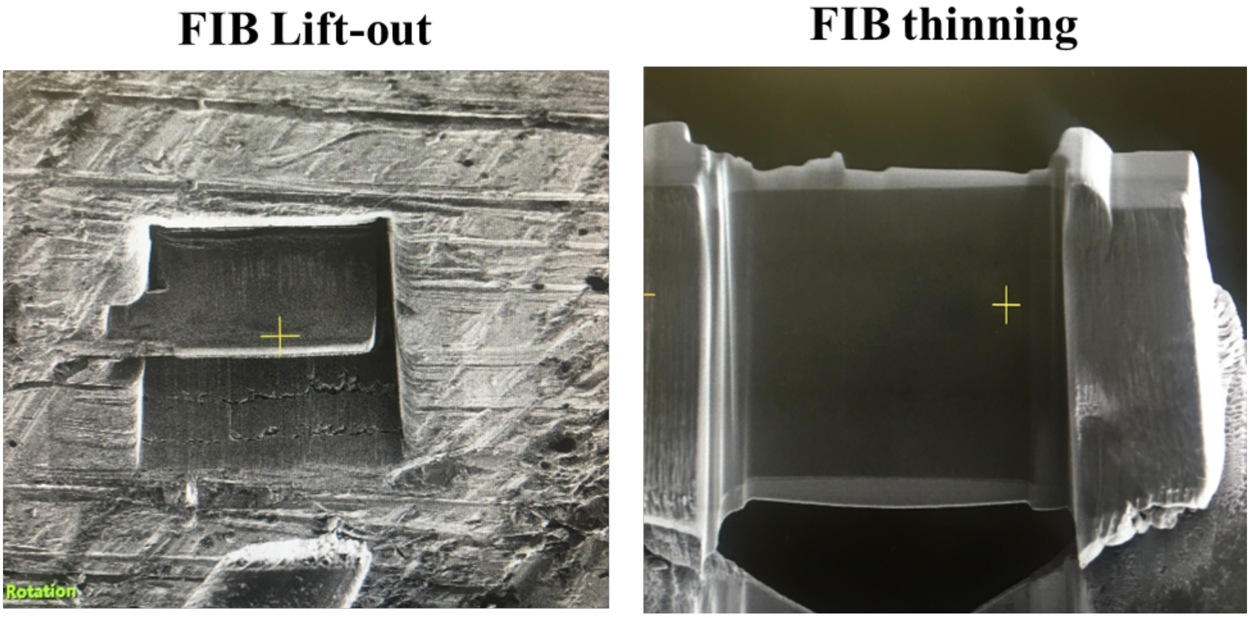

Fig. S4 Example of the FIB TEM sample preparation

TEM observation of the thin foils were then conducted in the JEOL F200 TEM operated at an accelerating voltage of 200 kV. In all cases, imaging was carried out in STEM mode so as to show the highest dislocation density.

TDA experiments

Hydrogen was electrochemically charged into the tensile test specimens using 1 g/L in an aqueous solution of 3 wt% NaCl and 0.3 wt% NH4SCN with a current of 10 $mA{\cdot}cm^{-2}$ for 48 h at room temperature (*44*).

To measure the concentrations of diffusible H in the H-charged specimens, thermal desorption analysis (TDA) was conducted using a gas chromatography system (Agilent Technologies 7890A) and a tubular furnace in a He gaseous atmosphere (*45*, *46*). TDA was conducted within 10 min after H charging. Continuous heating from room temperature to 700 ºC was used at a rate of 100 ºC h^{-1}, shown in Fig. S5. The hydrogen gas released from the specimen was analysed at 3 min intervals in a He carrier gas.

Hydrogen Charging Cell **TDA**

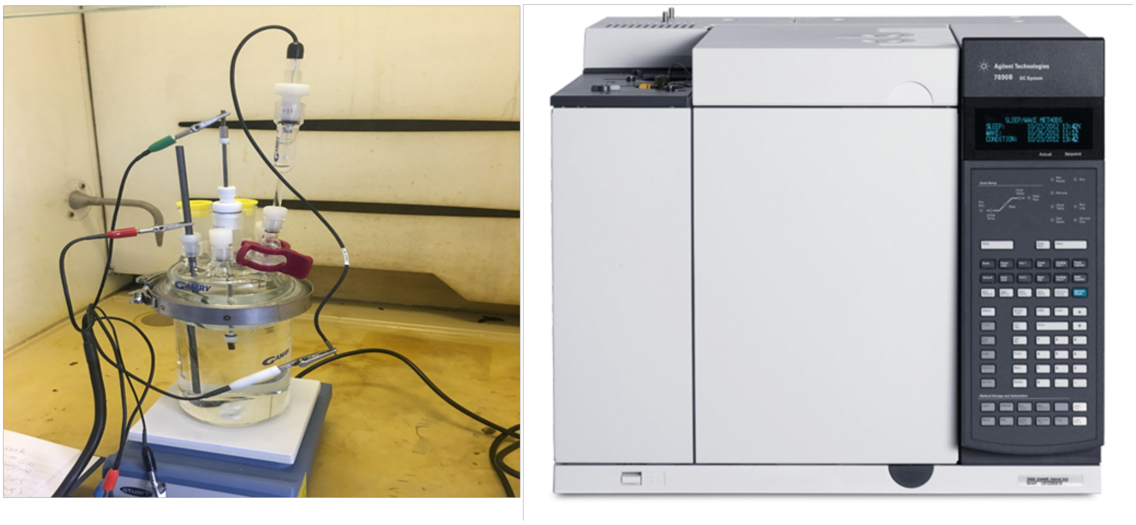

Fig. S5 the images for the hydrogen charging cell and TDA machine.
Photo Credit: Peng Gong, University of Sheffield.

Nanomegas experiments

TEM-based automated crystal orientation mapping (ACOM), developed by NanoMEGAS, uses selected area diffraction patterns to determine crystal orientation and allow for rapid collection of diffraction patterns (>200 per second) and reliable indexing of the phase and orientation of the crystal with a spatial resolution of 2 nm (*47*, *48*). This allows rapid collection of 2D maps that contain a wealth of information about the microstructure including grain size, grain boundary character and texture.

The ACOM patterns were obtained with a precession angle and frequency of 0.7° and 100 Hz respectively. In order to optimise collection time against pattern quality an exposure time of 40 ms per frame was used. Figure S6 shows typical examples of the precession electron diffraction (PED) pattern obtained, free from Kikuchi lines.

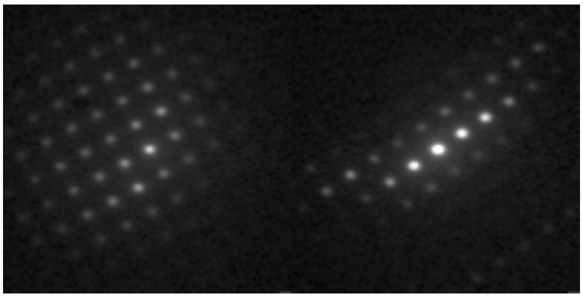

Fig. S6 Typical PED patterns obtained at 0.7° precession angle.
Photo Credit: Peng Gong, University of Sheffield.

A step size of 2.5 nm was used, with a total of 480 by 200 steps in the case of the Ti-Mo sample and 400 by 400 in the case of the V-Mo sample, for a total mapped area of 1.2 by 0.5 and 1.0 by 1.0 micrometres respectively. These areas were selected to ensure that all features of interest could be captured in a single map. The Martensite Twins were mapped using a step size of 5 nm with a total area of 2.0 by 2.0 microns (400 by 400 steps). This step size was used to balance image quality with collection time.

These maps were indexed using a simulated diffraction pattern for BCC Iron with lattice parameters of 2.861 nm. A total of 1326 different patterns were generated using the associated DiffGen 2 software. Patterns were generated with step count of 50.

REFERENCES AND NOTES

1. W. H. Johnson, On some remarkable changes produced in iron and steel by the action of hydrogen and acids. *Nature* **11**, 393 (1875).

2. G. S. Frankel, J. D. Vienna, J. Lian, J. R. Scully, S. Gin, J. V. Ryan, J. W. Wang, S. H. Kim, W. Windl, J. C. Du, A comparative review of the aqueous corrosion of glasses, crystalline ceramics, and metals. *npj Mater. Degrad.* **2**, 15 (2018).

3. J. P. Hanson, A. Bagri, J. Lind, P. Kenesei, R. M. Suter, S. Gradecak, M. J. Demkowicz, Crystallgraphic character of grain boundaries resistant to hydrogen-assisted fracture in Ni-base alloy 725. *Nat. Commun.* **9**, 3386 (2018).

4. H. C. Rogers, Hydrogen embrittlement of metals. *Science* **159**, 1057–1064 (1968).

5. J. Cairney, Atoms on the move-finding the hydrogen. *Science* **355**, 1128–1129 (2017).

6. H. H. Johnson, Hydrogen embrittlement. *Science* **179**, 228–230 (1973).

7. L. Grunberg, D. T. Jamieson, D. Scott, R. A. Lloyd, Hydrogen diffusion in water-accelerated rolling surface fatigue. *Nature* **188**, 1182–1183 (1960).

8. K. Ming, L. Li, Z. Li, X. Bi, J. Wang, Grain boundary decohesion by nanoclustering Ni and Cr separately in CrMnFeCoNi high-entropy alloys. *Sci. Adv.* **5**, eaay0639 (2019).

9. M. Seita, J. P. Hanson, S. Gradečak, M. J. Demkowjcz, The dual role of coherent twin boundaries in hydrogen embrittlement. *Nature Commun.* **6**, 6164 (2015).

10. T. Neeraj, R. Srinivasan, J. Li, Hydrogen embrittlement of ferritic steels: Observations on deformation microstructure, nanoscale dimples and failure by nanovoiding. *Acta Mater.* **60**, 5160–5171 (2012).

11. M. Koyama, C. C. Tasan, E. Akiyama, K. Tsuzaki, D. Raabe, Hydrogen-assisted decohesion and localized plasticity in dual-phase steel. *Acta Mater.* **70**, 174–187 (2014).

12. M. Wang, E. Akiyama, K. Tsuzaki, Effect of hydrogen on the fracture behavior of high strength steel during slow strain rate test. *Corros. Sci.* **49**, 4081–4097 (2007).

13. S. Wang, A. Nagao, P. Sofronis, I. M. Robertson, Hydrogen-modified dislocation structures in a cyclically deformed ferritic-pearlitic low carbon steel. *Acta Mater.* **144**, 164–176 (2018).

14. M. S. Chowdhury, W. Zheng, S. Kumari, J. Heyman, X. Zhang, P. Dey, D. A. Weitz, R. Haag, Dendronized fluorosurfactant for highly stable water-in-fluorinated oil emulsions with minimal inter-droplet transfer of small molecules. *Nat. Commun.* **10**, 4546 (2019).

15. E. Joonaki, J. Buckman, R. Burgass, B. Tohidi, Water *versus* asphaltenes; liquid–liquid and solid–liquid molecular interactions unravel the mechanisms behind an improved oil recovery methodology. *Sci. Rep.* **9**, 11369 (2019).

16. S. J. Kim, E. H. Hwang, J. S. Park, S. M. Ryu, D. W. Yun, H. G. Seong, Inhibiting hydrogen embrittlement in ultra-strong steels for automotive applications by Ni-alloying. *npj Mater. Degrad.* **3**, 12 (2019).

17. G. Williams, C. Kousis, N. McMurray, P. Keil, A mechanistic investigation of corrosion-driven organic coating failure on magnesium and its alloys. *npj Mater. Degrad.* **3**, 41 (2019).

18. T. Dieudonné, L. Marchetti, M. Wery, J. Chêne, C. Allely, P. Cugy, C. P. Scott, Role of copper and aluminum additions on the hydrogen embrittlement susceptibility of austenitic Fe–Mn–C TWIP steels. *Corros. Sci.* **82**, 218–226 (2014).

19. J. Lee, T. Lee, D.-J. Mun, C. M. Bae, C. S. Lee, Comparative study on the effects of Cr, V, and Mo carbides for hydrogen-embrittlement resistance of tempered martensitic steel. *Sci. Rep.* **9**, 5219 (2019).

20. Y.-S. Chen, D. Haley, S. S. A. Gerstl, A. J. London, F. Sweeney, R. A. Wepf, W. M. Rainforth, P. A. J. Bagot, M. P. Moody, Direct observation of individual hydrogen atoms at trapping sites in a ferritic steel. *Science* **355**, 1196–1199 (2017).

21. Y.-S. Chen, H. Lu, J. Liang, A. Rosenthal, H. Liu, G. Sneddon, I. McCarroll, Z. Zhao, W. Li, A. Guo, J. M. Cairney, Observation of hydrogen trapping at dislocations, grain boundaries, and precipitates. *Science* **367**, 171–175 (2020).

22. M. Hatano, M. Fujinami, K. Arai, H. Fujii, M. Nagumo, Hydrogen embrittlement of austenitic stainless steels revealed by deformation microstructures and strain-induced creation of vacancies. *Acta Mater.* **67**, 342–353 (2014).

23. B. A. Szost, R. H. Vegter, P. E. J. Rivera-Díaz-del-Castillo, Hydrogen-trapping mechanisms in nanostructured steels. *Metall. Mater. Trans. A* **44**, 4542–4550 (2013).

24. H. K. D. H. Bhadeshia, Prevention of hydrogen embrittlement in steels. *ISIJ Int.* **56**, 24–36 (2016).

25. R. A. Oriani, Hydrogen embrittlement of steels. *Ann. Rev. Mater. Sci.* **8**, 327–357 (1987).

26. S. K. Dwivedi, M. Vishwakarma, Hydrogen embrittlement in different materials: A review. *Int. J. Hydrog. Energy* **43**, 21603–21616 (2018).

27. M. Nagumo, Function of hydrogen in embrittlement of high-strength steels. *ISIJ Int.* **41**, 590–598 (2001).

28. K. Takasawa, R. Ikeda, N. Ishikawa, R. Ishigaki, Effects of grain size and dislocation density on the susceptibility to high-pressure hydrogen environment embrittlement of high-strength low-alloy steels. *Int. J. Hydrog. Energy* **37**, 2669–2675 (2011).

29. J. Song, W. A. Curtin, Atomic mechanism and prediction of hydrogen embrittlement in iron. *Nat. Mater.* **12**, 145–151 (2013).

30. N. R. Moody, Hydrogen effects on material behavior and corrosion deformation interactions: Proceedings of the international conference on hydrogen effects on material behavior and corrosion deformation interations: Held at Jackson Lake Lodge, Moran, Wyoming, September 22-26, 2002. *Eff. Hydrog. Behav. Mater.* 449–466 (2003).

31. L. P. Kubin, Y. Estrin, Evolution of dislocation densities and the critical conditions for the Portevin-Le Châtelier effect. *Acta Metal. et Mater.* **38**, 697–708 (1990).

32. Y. Estrin, L. S. Toth, A. Molinari, Y. Bréchet, A dislocation-based model for all hardening stages in large strain deformation. *Acta. Mater.* **46**, 5509–5522 (1998).

33. L. S. Toth, A. Molinari, Y. Estrin, Strain hardening at large strains as predicted by dislocation based. *J. Eng. Mater. Technol.* **124**, 71–77 (2002).

34. F. Maresca, W. A. Curtin, Theory of screw dislocation strengthening in random BCC alloys from dilute to "High-Entropy" alloys. *Acta Mater.* **182**, 144–162 (2019).

35. E. I. Galindo-Nava, J. Sietsma, P. E. J. Rivera-Díaz-del-Castillo, Dislocation annihilation in plastic deformation: II. Kocks–Mecking Analysis. *Acta Mater.* **60**, 2615–2624 (2012).

36. E. I. Galindo-Nava, P. E. J. Rivera-Díaz-del-Castillo, A thermodynamic theory for dislocation cell formation and misorientation in metals. *Acta Mater.* **60**, 4370–4378 (2012).

37. H. Fu, P. E. J. Rivera-Díaz-del-Castillo, A unified theory for microstructural alterations in bearing steels under rolling contact fatigue. *Acta Mater.* **155**, 43–55 (2018).

38. S.-X. Li, P.-C. Zhao, Y.-S. Su, S.-R. Yu, Investigation of the root cause of subsurface damage in wind turbine gearbox bearings. *Tribol. Int.* **102**, 546–554 (2016).

39. L. Schlapbach, A. Züttel, P. Gröning, O. Gröning, P. Aebi, Hydrogen for novel materials and devices. *Appl. Phys. A* **72**, 245–253 (2001).

40. S. G. Hashemi, B. Eghbali, Analysis of the formation conditions and characteristics of interphase and random vanadium precipitation in a low-carbon steel during isothermal heat treatment. *Int. J. Miner. Metall. Mater.* **25**, 339–349 (2018).

41. A. Ikeda, T. Kaneko, Y. Ando, On the evaluation method of sulfide stress cracking susceptibility of carbon and low alloy steels. *Corros. Sci.* **27**, 1099–1115 (1987).

42. J. A. Beavers, G. H. Koch, Limitations of the slow strain rate test for stress corrosion cracking testing. *Corrosion* **48**, 256–264 (1992).

43. R. M. Langford, A. K. Petford-Long. Preparation of transmission electron microscopy cross-section specimens using focused ion beam milling. *J. Vac. Sci. Technol. A.* **19**, 2186–2193 (2001).

44. F. G. Wei, K. Tsuzaki, Hydrogen absorption of incoherent TiC particles in iron from environment at high temperatures. *Metall. Mater. Trans. A Phys. Metall. Mater. Sci.* **35A**, 3155–3163 (2004).

45. K. Takai, J. Seki, Y. Homma, Observation of trapping sites of hydrogen and deuterium in high-strength steels by using secondary ion mass spectrometry. *Mater. Trans. JIM* **36**, 1134–1139 (1995).

46. K. Takai, G. Yamauchi, M. Nakamura, and M. Nagumo, Hydrogen trapping characteristics of cold-drawn pure iron and eutectoid steel evaluated by thermal desorption spectrometry. *J. JPN I. Met.* **62**, 267–75 (1998).

47. D. Viladot, M. Véron, M. Gemmi, F. Peiró, J. Portillo, S. Estradé, J. Mendoza, N. Llorca-Isern, S. Nicolopoulos, Orientation and phase mapping in the transmission electron microscope using precession-assisted diffraction spot recognition: State-of-the-art results. *J. Microsc.* **252**, 23–34 (2013).

48. E. F. Rauch, M. Véron, Automated crystal orientation and phase mapping in TEM. *Mater. Charact.* **98**, 1–9 (2014).
